# Impact of perinatal and repeated maternal common mental disorders on educational outcomes of primary school children in rural Ethiopia: population-based cohort study

**DOI:** 10.1192/bjo.2019.69

**Published:** 2019-10-07

**Authors:** Habtamu Mekonnen, Girmay Medhin, Mark Tomlinson, Atalay Alem, Martin Prince, Charlotte Hanlon

**Affiliations:** Assistant Professor, Department of Psychiatry, School of Medicine, College of Health Sciences, Addis Ababa University; and Department of Psychology, College of Education and Behavioural Sciences, Jimma University, Ethiopia; Associate Professor, Aklilu-Lemma Institute of Pathobiology, Addis Ababa University, Ethiopia; Professor, Department of Psychology, Stellenbosch University, South Africa; Professor, Department of Psychiatry, School of Medicine, College of Health Sciences, Addis Ababa University, Ethiopia; Professor, Assistant Principal, Epidemiological Psychiatry, King's Global Health Research Institute, King's College London, UK; Reader, Centre for Global Mental Health, Health Service and Population Research Department, Institute of Psychiatry, Psychology and Neuroscience, King's College London, UK; Department of Psychiatry, School of Medicine, College of Health Sciences, Addis Ababa University; and Centre for Innovative Drug Development and Therapeutic Trials for Africa (CDT-Africa), College of Health Sciences, Addis Ababa University, Ethiopia

**Keywords:** Education, absenteeism, mental health, postnatal depression, sub-Saharan Africa

## Abstract

**Background:**

There have been no studies from low- or middle-income countries to investigate the long-term impact of perinatal common mental disorders (CMD) on child educational outcomes.

**Aims:**

To test the hypothesis that exposure to antenatal and postnatal maternal CMD would be associated independently with adverse child educational outcomes in a rural Ethiopian.

**Method:**

A population-based birth cohort was established in 2005/2006. Inclusion criteria were: age between 15 and 49 years, ability to speak Amharic, in the third trimester of pregnancy and resident of the health demographic surveillance site. One antenatal and nine postnatal maternal CMD assessments were conducted using a self-reporting questionnaire, validated for the local use. Child educational outcomes were obtained from the mother at *T*_1_ (2013/2014 academic year; mean age 8.5 years) and from school records at *T*_2_ (2014/2015 academic year; mean age 9.3 years).

**Results:**

Antenatal CMD (risk ratio (RR) = 1.06, 95% CI 1.05–1.07) and postnatal CMD (RR = 1.07, 95% CI 1.06–1.09) were significantly associated with child absenteeism at *T*_2_. Exposure to repeatedly high maternal CMD scores in the preschool period was not associated with absenteeism after adjusting for antenatal and postnatal CMD. Non-enrolment at *T*_1_ (odds ratio 0.75, 95% CI 0.62–0.92) was significantly but inversely associated with postnatal maternal CMD. There was no association between maternal CMD and child academic achievement or drop-out.

**Conclusions:**

Our findings support the hypothesis of a critical period for exposure to maternal CMD for adverse child outcomes and indicate that programmes to enhance regular school attendance in low-income countries need to address perinatal maternal CMD.

**Declaration of interest:**

None.

Women of childbearing age are at increased risk of common mental disorders (CMD),^1^ mainly depression and anxiety.^2,3^ Postnatal depression has an estimated prevalence of 13%^4^ in high-income countries (HICs) and 19.8% in low- and middle-income (LMICs) countries.^5^ The negative impact of maternal CMD extends beyond the individual woman to affect family, work, social relationships and interactions with the child.^1,4^ Children may be particularly vulnerable to the negative effects of maternal CMD^1^ as they are dependent on the mother for both physical and psychological needs. In HICs there have been studies indicating that the perinatal period represents a critical period for exposure to maternal CMD;^6,7^ whereas others argue that recurrent exposure to adversity is more important than exposure at a specific time.^8^ In LMICs, there have been short-term studies of the impact of ante- and postnatal CMD on infant growth, health and early child development,^1,9–11^ but we are not aware of any that have sought to disaggregate the effect of perinatal CMD from subsequent exposure to maternal CMD. Furthermore, few studies have investigated the impact of maternal CMD beyond infancy, so that little evidence is available to understand the longer-term impact on child educational outcomes.^12,13^

The aim of this study was to investigate the impact of perinatal (antenatal and postnatal) and preschool maternal CMD upon educational outcomes of children. We hypothesised that children exposed to maternal antenatal CMD, postnatal CMD and subsequent repeated high maternal CMD score episodes in the pre-school period would have lower school enrolment, lower academic achievement, higher absence and higher drop-out from primary school.

## Method

### Study design

The study was an extension of a population-based birth cohort, the Child outcomes in relation to Maternal Mental Illness in Ethiopia (C-MaMiE) study. A total of 1065 pregnant women were recruited in 2005–2006 and have been followed up, together with the index child, to date. In the current study, we examined the exposure of maternal CMD measured repeatedly since the inception of the cohort in relation to the birth date of the child. Educational outcomes of children were measured at two time points: *T*_1_ (2013/2014 academic year, child mean age of 8.5 years (s.d. = 0.3) and *T*_2_ (2014/2015 academic year, mean age of 9.3 years, s.d. = 0.3).

### Study setting

The C-MaMiE cohort was established within the Health and Demographic Surveillance Site (HDSS)^14^ in Butajira, Gurage Zone, Southern Nations Nationalities and Peoples' Region of Ethiopia. Butajira is located 135 km south of the capital Addis Ababa, is predominantly rural and is notable for the diversity of ethnicities and languages in the population. The HDSS has nine subdistricts with different ecological zones (low and highlands) and one urban administration in Butajira town. Butajira is densely populated and livelihoods are based on mixed farming of staples, such as maize and false banana, and cash crops, such as khat and chilli peppers.

#### Context for education

Ethiopia is striving for complete primary education coverage, although national figures from the 2015/2016 academic year indicate that only 85.5% have enrolled currently, with 10.1% drop-out and 6.7% grade repetition.^15^ The official age for school enrolment is 7 years. Primary education lasts for 8 years (age group 7–14 years) with two cycles: basic (grades 1–4) and general education (grades 5–8). Families are expected to cover the costs of school uniforms, food and exercise books; otherwise, education is free for all Ethiopians.^16^ Except for one regional examination at the completion of grade 8, the academic performance of students is assessed by the class teacher using non-standardised tests. In the first cycle of primary education, children are taught and evaluated by a single teacher following the ‘self-contained class’ concept.^16^

### Study participants

At the inception of the C-MaMiE cohort, a population-based sample of 1065 women was recruited out of 1234 eligible women (86.3%) meeting inclusion criteria of age between 15 and 49 years, ability to communicate in Amharic, a resident of the HDSS and in the third trimester of pregnancy. The women and the child born from the index pregnancy have been assessed repeatedly over time. Ten time-point assessments were conducted starting in pregnancy and at 2, 12, 30, 36, 42, 48, 60, 78 and 102 months of age of the child in the postnatal period, see supplementary Fig. 1 available at https://doi.org/10.1192/bjo.2019.69.

### Measures

#### Educational outcomes

The primary educational outcomes for this study (school enrolment, absenteeism, drop-out and academic performance) were selected based on their contextual relevance, given that (a) key bottlenecks to academic success occur at the stages of school enrolment (although working towards 100% coverage, the most recent estimates of school enrolment in Ethiopia are 85.5%), regular attendance and retention in school in this setting, and (b) that regular school attendance has been shown to have important socialisation benefits, regardless of the impact on academic achievement.^17,18^ All outcomes were measured in relation to academic years rather than the birth date of the children as shown in supplementary Fig. 1. They were measured as follows.

- Enrolment: each mother was asked whether the child had ever been enrolled in school by *T*_1_.
- Absenteeism: total number of days of absence was obtained from daily school attendance records at *T*_2_.
- School drop-out: students who had enrolled at the beginning of the academic year (September) but who had dropped out of school before the end of the academic year (June) were deemed to have dropped out; for students who were absent from school for a period of time during the school year, but who were attending school at the end of the year, the child was classed as absent and not dropped out. Children who drop-out of school can be re-enrolled and, therefore, are at risk of dropping out again in the subsequent academic year. For *T*_1_ we obtained the information from the mother, but for *T*_2_ we extracted the information from school records.
- Academic achievement: the teacher-reported averaged grade point over two semesters of the Ethiopian school year and this was obtained from school records at *T*_2_. Teacher assessments of academic achievement are composite and non-standardised, based on continuous assessment of mastery of content, class participation and interaction, conduct, homework, progress over time and school attendance.

#### Primary exposure

##### Maternal CMD

This was measured using the World Health Organization 20-item version of the Self-Reporting Questionnaire (SRQ-20) in pregnancy and at all nine postnatal time points until the child was on average 8.5 years.^19^ The SRQ-20 items ask about the presence or absence of depressive, anxiety and somatic symptoms in the preceding 1 month (answered ‘yes’ or ‘no’). The SRQ-20 has been validated for perinatal women in this rural Ethiopian population.^20^ Repeated high maternal CMD scores were generated as the count of time points after the 2-month postnatal time point when the woman scored ≥6.

#### Potential confounding factors

Measures of potential confounding factors were used from the following time points depending on the hypothesis: (a) model 1 (exposure of antenatal CMD): pregnancy time point, (b) model 2 (exposure of postnatal CMD): postnatal 2-month time point, and (c) model 3 (exposure of repeated high maternal CMD scores): 60 months postnatal time point.

##### Stressful life events

An adapted version of the 12 item List of Threatening Experiences^21^ scale was used to measure stressful life events over the pregnancy (antenatal time point) and preceding 6 months for the postnatal assessments.

##### Socioeconomic status

Self-report of the following proxy indicators of socioeconomic status were measured: current roof material, the experience of hunger in the preceding month because of lack of food or money, and the existence of emergency resources in times of crisis.

##### Paternal substance use

A report of the frequency of paternal alcohol and/or khat use was obtained from the woman.

##### Demographic characteristics

Literacy level of both parents, age of the mother, marital status, birth order and gender of the child were obtained from self-report of the woman.

##### Child nutritional status

Height measures were carried out by trained project data collectors using a stadiometer with a movable headpiece. Using the World Health Organization reference population,^22^ height-for-age *z-*scores were calculated using World Health Organization Anthro software.^23^

### Data management

#### Data collection procedure

To ensure privacy, confidentiality and her preference, all interviews with the women were carried out in the woman's home or surrounding area. The project data collectors had all completed high-school education, and were experienced in conducting interviews and in the use of the study measures. At each time point, they received an additional 3 days of refresher training on the use of newly added instruments. The questionnaires were piloted before commencing data collection and discrepancies in ratings were discussed to ensure that the data collectors had a common understanding.

#### Maintaining data quality

Supervisors and a field coordinator monitored the data-collection process and performed quality checks on a random sample of evaluations. Data-entry clerks double entered data with EpiData version 3.1^24^ on the day of data collection, where possible. Any identifiable information about the respondent was kept securely and separately from the assessment data and a code number was used to ensure confidentiality.

### Statistical analyses

A hypothesis-driven analysis was conducted using Stata version 12^25^ to examine the association of maternal CMD in model 1 antenatally, and model 2 at 2 months postnatal using SRQ-20 total score, and in model 3 repeated high maternal CMD scores (as previously defined) with educational outcomes. First, we conducted unadjusted logistic regression for school non-enrolment and drop-out (binary outcomes), zero-inflated Poisson regression for absenteeism (count data, with excess zeroes) and linear regression for academic achievement (continuous, normally distributed). We then carried out two stages of multivariable analysis; first a model containing each primary exposure adjusted for all potential confounders identified *a priori*, and finally we ran a model containing antenatal, postnatal and repeated high CMD scores and all potential confounders at the 60-month time point in the same model. Estimates of associations were presented with their corresponding 95% confidence intervals. The study has been reported according to the STROBE reporting checklist.

### Ethical considerations

The authors assert that all procedures contributing to this work comply with the ethical standards of the relevant national and institutional committees on human experimentation and with the Helsinki Declaration of 1975, as revised in 2008. All procedures involving human participants were approved by the Institutional Review Board of the College of Health Sciences, Addis Ababa University, Ethiopia (reference number 082/13/psy) and the Research Ethics Committee of King's College London, UK (reference number PNM/13/14-92). Written or verbal informed consent was obtained from each woman for her own and her child's participation in the study as well and to access school records of the child. Verbal consent was witnessed and formally recorded. For the anthropometric assessment assent was obtained from the child. Any woman who presented with high CMD symptoms and suicidal ideation was supported to seek care at the psychiatric unit at Butajira Hospital, with the project covering treatment and transportation costs.

## Results

**Fig. 1:**
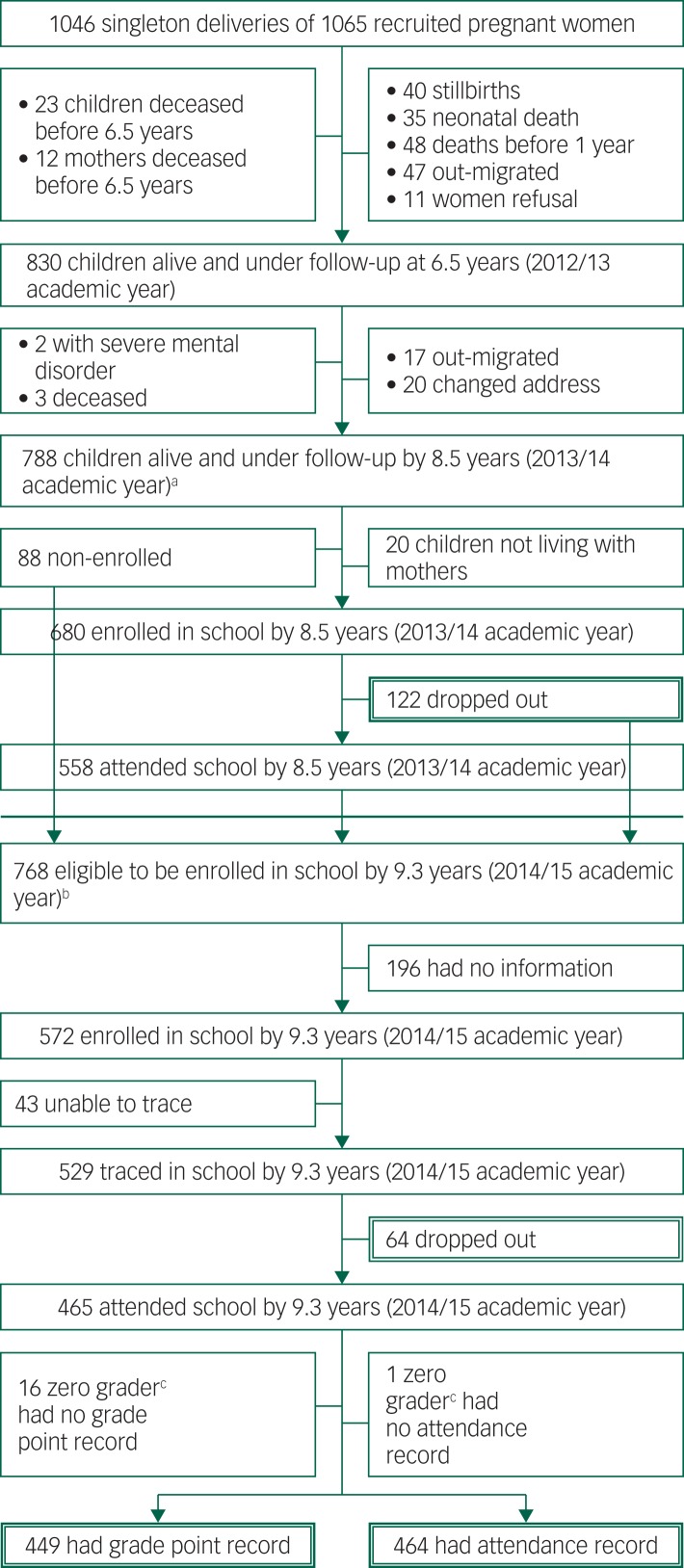
Flow chart of Child outcomes in relation to Maternal Mental Illness in Ethiopia cohort participants.

At the latest maternal CMD exposure time point before *T*_1_, when the children were a mean of 6.5 years (s.d. = 0.03), a total of 830 (77.9%) mother–child dyads participated in the study. See Table 1 for characteristics of participants. At the latest exposure time point before *T*_2_, when the children were a mean of 8.5 years (s.d. = 0.3, minimum 7.90, maximum 9.17 years), 788 (74.0%) participated in the study. Most loss to follow-up occurred early in the cohort as a result of stillbirth (*n* = 40), neonatal death (*n* = 35) and post-neonatal infant mortality (*n* = 48 by 12 months). A further 12 mothers and 23 children died before the age of 6.5 years. A total of 47 women had out-migrated by 6.5 years. Maternal refusal was low (*n* = 11) (Fig. 1).

a. Assessment time point 1. b. Assessment time point 2. c. Zero graders means a child is enrolled in school but has no formal academic records.

**Table 1:** Summary of exposure variables at the beginning of educational follow-up

Characteristics measured at 6.5 years (total *n* = 830)	Value
Maternal age, years: mean (s.d.)	34.1 (6.3)
Maternal literacy, *n* (%)	
Literate	120 (14.5)
Non-literate	710 (85.5)
Paternal literacy (*n* = 801), *n* (%)	
Literate	512 (63.9)
Non-literate	289 (36.1)
Marital status, *n* (%)	
Monogamous	663 (79.9)
Polygamous	138 (16.6)
Divorced, widowed or separated	29 (3.5)
Had hunger in preceding month, *n* (%)	
Yes	40 (4.8)
No	790 (95.2)
Had emergency resources, *n* (%)	
Yes	506 (61.0)
No	324 (39.0)
Roof material, *n* (%)	
Thatched	565 (68.1)
Corrugated iron	265 (31.9)
Father's khat use (*n* = 800), *n* (%)	
Less than weekly	620 (77.5)
Weekly	180 (22.5)
Father's alcohol use (*n* = 800), *n* (%)	
Less than weekly	681 (85.1)
Weekly	119 (14.9)
Negative life event in the past 6 months (*n* = 824), *n* (%)	
No time point	661 (80.2)
One time point	132 (16.0)
More than one time point	31 (3.8)
Maternal mental health, *n* (%)	
High SRQ-20	37 (4.5)
Low SRQ-20	793 (95.5)
Childbirth order, *n* (%)	
First	112 (13.5)
Middle or last	718 (86.5)
Sex of the child, *n* (%)	
Girl	408 (49.2)
Boy	422 (50.8)
Child stunting (*n* = 820), *n* (%)	
Non-stunted	568 (69.3)
Stunted	252 (30.7)

SRQ, Self-Reporting Questionnaire.

There was no significant difference between those lost to follow-up and those remaining in the study in terms of demographic, socioeconomic and mental health status. Children for whom educational information was not available did not differ significantly on maternal CMD, socioeconomic status or birth order, but were more likely to have been boys and to have been stunted growth. See supplementary Tables 1 and 2.

### Maternal mental health

The median score on the SRQ (maternal CMD) was 2 (interquartile range (IQR) = 0–4) at the antenatal assessment and 1 (IQR = 0–2) at 2-months postnatal. In terms of repeated high maternal CMD scores from 12 to 102 months: 607 (73.1%) women had no high scores (≥6), 148 (17.8%), scored high at one time point and 75 (9.0%) had a high score at more than one time point. Of the 84 women who had repeated high CMD scores in the preschool period, 34.7% (*n* = 26) also scored high at the antenatal time point and 22.7% (*n* = 17) scored high at the 2-month postnatal time point.

### Child education outcomes

At *T*_1_ (2013/14 academic year), 88 children had still not enrolled in school (11.2%), and a further 122 (17.9%) had enrolled but subsequently dropped out. At *T*_2_, during the 2014/2015 academic year, 64 children (12.1%) dropped out. The median number of days of absence was 5 (IQR = 2–11), with 82 children (17.6%) recorded as having no absence. The mean grade point was 62.6 out of 100 (s.d. = 9.5).

### Association between maternal CMD and child education outcomes

Postnatal CMD (at 2 months) was associated inversely with child school non-enrolment (adjusted OR = 0.75, 95% CI 0.62–0.92) at *T*_1_, but there was no association between antenatal or repeated high maternal CMD scores and non-enrolment.

Antenatal CMD (adjusted risk ratio (RR) = 1.06, 95% CI 1.05–1.07) and postnatal CMD (adjusted RR = 1.21, 95% CI 1.16–1.26) were independently and significantly associated with absenteeism, but there was no association between repeated high maternal CMD scores and absenteeism in the multivariable model containing antenatal and postnatal CMD. Exposure to maternal CMD at any time point was not associated with either school drop-out or the academic achievement of the child (Tables 2 and 3).

**Table 2:** Impact of maternal common mental disorders (CMD) on child educational outcomes at time point 1 (2013/14 academic year)

Models, primary exposure	Odds ratio (95% CI)	
	Association of maternal CMD and school non-enrolment at *T*_1_ (*n* = 768)	Association of maternal CMD & school drop-out at *T*_1_ (*n* = 680)
Unadjusted models		
Antenatal CMD: total score on SRQ-20	1.03 (0.97–1.10)	1.05 (0.99–1.11)
Postnatal CMD (at 2 months): total score on SRQ-20	**0.85 (0.73–0.99)**	1.05 (0.97–1.13)
Repeated high maternal CMD scores:^a^ sum of all postnatal SRQ high score (≥ 6) excluding 2 months	1.06 (0.76–1.36)	1.22 (0.96–1.56)
Maternal CMD exposures in separate multivariable models adjusted^b^ for confounders	
Antenatal	0.97 (0.88–1.07)	1.04 (0.96–1.12)
Postnatal (2 months)	**0.75 (0.62–0.92)**	1.00 (0.90–1.11)
Repeated high maternal CMD scores (without 2 months)	0.98 (0.64–1.51)	1.21 (0.89–1.63)
Fully adjusted^c^ model including all maternal CMD exposures in the same model		
Antenatal	1.01 (0.91–1.11)	1.03 (0.95–1.11)
Postnatal (2 months)	**0.74 (0.60–0.91)**	0.94 (0.85–1.05)
Repeated high maternal CMD scores (without 2 months)	1.15 (0.71–1.84)	1.27 (0.90–1.79)

*T*_1_, assessment time point 1; SRQ, Self-Reporting Questionnaire.

a. Repeated high maternal CMD scores from 12 to 60 and 12 to 78 months for enrolment and drop-out outcomes respectively.

b. Parental characteristics, including (maternal age, marital status, maternal and paternal level of literacy), socioeconomic status (SES), paternal substance use, negative life event, child gender, birth order and child nutritional status.

c. Parental characteristics, including (maternal age, marital status, maternal and paternal level of literacy), SES, paternal substance use, negative life event, child gender, birth order and child nutritional status, antenatal and postnatal CMD.

Bold signifies significant results at *P* < 0.05.

**Table 3:** Impact of maternal common mental disorders (CMD) on child educational outcomes at time point 2 (2014/15 academic years)

Models, primary exposure	Association of maternal CMD and school absenteeism at *T*_2_, count ratio (95% CI) (*n* = 464)	Association of maternal CMD and school drop-out at *T*_2_, odds ratio ((95% CI) (*n* = 529)	Association of maternal CMD and academic achievement at *T*_2_, β (95% CI) (*n* = 449)
Unadjusted			
Antenatal CMD total score on SRQ-20	**1.05 (1.04 to 1.06)**	1.06 (0.99 to 1.14)	0.004 (−0.26 to 0.27)
Postnatal CMD (at 2 months) total score on SRQ-20	**1.06 (1.05 to 1.07)**	1.00 (0.89 to 1.12)	0.08 (−0.29 to 0.44)
Repeated high maternal CMD scores^a^	**1.14 (1.11 to 1.18)**	1.21 (0.94 to 1.55)	−0.09 (−1.08 to 0.89)
Maternal CMD exposures in separate multivariable models^b^			
Antenatal	**1.06 (1.05 to 1.07)**	1.03 (0.94 to 1.13)	0.08 (−0.26 to 0.41)
Postnatal (2 months)	**1.07 (1.06 to 1.09)**	0.99 (0.86 to 1.14)	0.34 (−0.13 to 0.82)
Repeated high maternal CMD scores (without 2 months)	**1.21 (1.16 to 1.26)**	1.06 (0.78 to 1.44)	0.05 (−1.15 to 1.24)
Fully adjusted^c^ model including all maternal CMD exposures in the same model			
Antenatal	**1.05 (1.04 to 1.06)**	1.06 (0.96 to 1.16)	−0.10 (−0.46 to 0.25)
Postnatal (2 months)	**1.04 (1.03 to 1.06)**	0.91 (0.78 to 1.06)	0.26 (−0.23 to 0.75)
Repeated high maternal CMD scores^a^	1.05 (0.99 to 1.11)	1.08 (0.73 to 1.57)	−0.14 (−1.51 to 1.23)

*T*_2_, assessment time point 2; SRQ, Self-Reporting Questionnaire.

a. Repeated high maternal CMD scores from 12 to 102 months.

b. Parent's characteristics, including (maternal age, marital status, maternal and paternal level of literacy), socioeconomic status (SES), paternal substance use, negative life event, child gender, birth order and child nutritional status.

c. Parental characteristics, including (maternal age, marital status, maternal and paternal level of literacy), SES, paternal substance use, negative life event, child gender, birth order and child nutritional status, antenatal and postnatal CMD.

Bold signifies significant results at *P* < 0.05.

## Discussion

### Main findings

In this longitudinal prospective cohort study from a rural population in Ethiopia, antenatal and postnatal maternal CMD were found to have an independent significant association with school absenteeism at *T*_2_. Exposure to repeated high maternal CMD scores in early childhood was not associated with absenteeism after adjusting for antenatal and postnatal CMD. Postnatal CMD was significantly, but inversely, associated with school non-enrolment. There was no association between exposure to maternal CMD at any time point and either academic achievement or school drop-out.

### Strengths and limitations

To the best of our knowledge, this is the first population-based study from sub-Saharan Africa to have investigated the effect of perinatal CMD beyond infancy and examined the impact on educational outcomes of children. Furthermore, our study is the first of its kind from a LMIC to test the hypothesis that the perinatal period represents a critical period for exposure to maternal mental health problems. Strengths of the study are the population-based, prospective design with high follow-up rates and repeated measures of maternal CMD and confounding factors using culturally validated measures.

Nonetheless, there were also limitations. The exposure of ‘repeated high maternal CMD scores’ was generated from high scores at each assessment time point, but did not capture longitudinal exposure to maternal CMD between the time points. Absenteeism was extracted from data collected routinely by schools, which may not be accurate, although we expect that this would not lead to differential classification of students and would just reduce the power to detect an association. A teacher-reported (composite and non-standardised) measure of academic achievement was used. This composite measure may be more ecologically valid and tied to the day-to-day routine of teaching and learning than narrowly focused assessments of content mastery but this approach may increase measurement error. We were not able to adjust for child illness, although we did measure height-for-age which provides a proxy indicator of chronic ill health. We relied on proxy indicators of socioeconomic status. Although the socioeconomic status indicators have been developed for the population under study, they relied on self-report of the women and may not have been sufficiently comprehensive, thus raising the possibility of residual confounding.

### Comparison with findings from other studies and interpretation of findings

Evidence for the antenatal period as a critical time for exposure to maternal CMD in terms of later child outcomes is accumulating. In a large birth cohort study from the UK, an independent and significant association was observed between maternal anxiety at a gestational age of 32 weeks and an increased risk of child hyperactivity at the age of 4 years.^26^ Potential mechanisms were also explored: anxiety in late pregnancy was found to be associated with hypothalamic–pituitary axis function in the child, particularly the waking cortisol level of the child, which is responsible for orchestrating the stress response and has been implicated in development of emotional disorders.^27^ In HICs, a delayed effect of antenatal maternal CMD on the temperament of child has also been found.^28^ Our finding of a significant association between antenatal maternal CMD and later school absenteeism could, therefore, be linked with child temperament and child behavioural and emotional disorders. In support of this, we previously found an independent, prospective association between preschool child emotional and behavioural disorder and school absenteeism in an earlier analysis using an expanded C-MaMiE cohort. Further studies are required to elucidate the mechanisms mediating this association.

Our finding of a significant association between postnatal CMD and school absenteeism is supportive of the critical period hypothesis. A number of earlier studies from HICs^6,7^ and LMICs^12,29,30^ have shown an association between postnatal maternal CMD and child development. This association appears to be mediated by effects of postnatal CMD on the infant–mother relationship, which is predicted to lead to enduring effects on the child.^1^ Maternal responsiveness to the physical and psychological needs of an infant has a vital role in the development of secure attachment between the mother and infant.^7,31^ Maternal postnatal CMD was found to be associated with insecure attachment in a South African community-based study.^11,31^ Although adjustment for concurrent maternal sensitivity led to loss of a significant association in the South Africa study, this may reflect that postnatal depression led to changes in the mother–infant relationships that endured even when maternal depression resolved. Insecure attachment may, therefore, play a role in any enduring effect of postnatal CMD on child outcomes such as absenteeism.

Postpartum maternal CMD was found to predict lower academic performance of children in Barbados^13^ and in high-income settings;^6^ however, the result was not replicated in our study. Associations with academic performance in these previous studies have been mediated through associations between postnatal CMD and child cognitive development. However, in the Ethiopia C-MaMiE cohort we found no association between maternal CMD and child development.^32^ A previous study in Ethiopia also failed to replicate findings from Barbados of a specific adverse effect of early-life child malnutrition on academic performance of children in Barbados,^12^ with postnatal malnutrition no longer associated with later child development after current nutritional status was taken into account. Our null finding may be also because of the limitations of our measure of academic achievement, which was reliant on non-standardised teacher ratings. However, in this low-income setting, the effect of poverty on cognitive development and high maternal non-literacy may overwhelm any effects of maternal CMD. Substitute caregivers may compensate for a mother with depression and, therefore, limit the impact of maternal CMD on the child's learning environment. In contrast, absenteeism is likely to be related to more than cognitive development, also incorporating interpersonal functioning and confidence to separate from the mother, which are affected by security of attachment and may be more strongly associated with exposure to postnatal CMD.^11^

We did not find any association between exposure to repeated high maternal CMD scores in early childhood and any of the educational outcomes. This is in contrast to studies from Australia, a high-income country, where chronicity of exposure to maternal CMD was found to be associated with higher levels of child emotional and behavioural problems and lower vocabulary scores at later ages.^33^ Furthermore, our finding is at odds with the conceptualisation that child development is influenced by repeated exposure to developmental adversity.^1^ Our negative finding might have arisen because fewer than 10% of women had high CMD scores at more than one postnatal time point. This indicates that most children could have experienced extended periods of their early childhood when their mother did not have high CMD symptoms. Maternal resilience to mental health problems is an important area of focus for future studies.

The lack of association between any measure of maternal CMD (antenatal, postnatal or repeated high CMD scores) and school drop-out is in contrast to the study from HICs^34^ and our earlier report of an association between maternal CMD and school drop-out.^35^ The most likely explanation is that we were underpowered in this current analysis, which was performed on a subsample of the extended C-MaMiE cohort for whom longitudinal data from antenatal exposure onwards were available. Furthermore, for repeated high CMD scores, we dichotomised the SRQ-20 at each time point, which may also have decreased power to detect an effect. We would expect that absenteeism would be on the pathway to drop-out and that we would see similar patterns of association with maternal CMD. The significant but inverse association between postnatal maternal CMD and child school non-enrolment was unexpected, and we do not have an explanation for this finding. It is possible that this association was observed by chance (given the small numbers of children non-enrolled in school) or was affected by other unmeasured factors. This finding requires replication and further investigation.

### Implications

There is increasing research evidence of the importance of early investment in children to achieve optimal development, education and economic success. Indeed, for optimal child outcomes, the health of the mother is understood to be critical even before she is pregnant, as well as continuing through pregnancy and into the postnatal period. Our study indicates that there also needs to be a focus on maternal mental health, both in pregnancy and the early postnatal period, to optimise child educational outcomes. This has an important bearing on achievement of the Sustainable Development Goal seeking to achieve quality education for all children (goal 4).

In a LMIC, regular school attendance is not just the best way to learn, but frequently the only way to learn the taught topics, as there are limited learning resources at home or in the community to compensate for missed classes. For programmes aimed at enhancing regular school attendance, our study indicates that the perinatal mental health of women needs to be optimised. The high rates of health service contact of antenatal women provides an opportunity for intervention. The World Health Organization Mental health Gap Action Programme and other initiatives to task-share mental healthcare^36^ seek to equip maternal and primary healthcare workers to detect and treat CMD and other priority mental disorders. The Mental health Gap Action Programme intervention guide emphasises the importance of enhancing social support networks and addressing social stressors to address mental health problems, alongside evidence-based interventions. There is accumulating evidence for the efficacy of psychosocial interventions delivered by non-specialists to women with perinatal CMD, although most studies are from well-resourced settings in middle-income settings and none have examined the impact on child educational outcomes.^37^

In conclusion, our findings support the hypothesis of a critical period exposure to maternal CMD for adverse child outcomes, although further definitive evidence is required. Our findings indicate that programmes aimed at enhancing regular school attendance need to address maternal CMD from pregnancy onwards.

## Supplementary material

For supplementary material accompanying this paper visit http://dx.doi.org/10.1192/bjo.2019.69. click here to view supplementary material
