# Supplementary material for: Impact of perinatal and repeated maternal common mental disorders on educational outcomes of primary school children in rural Ethiopia: population-based cohort study
Source: BJPsych Open. 2019 Oct 7;5(6):e87. doi: 10.1192/bjo.2019.69 (PMC6788221; doi:10.1192/bjo.2019.69)
Supplement: Supplementary file 1 [file S2056472419000693sup001.docx]

# Supplementary Fig. 1: Time points of assessment

C-MaMiE cohort

Assessment time points of C-MaMiE cohort

Pregnancy

2 m

12 m

30m

36m

60m

48m

42m

102m

78m

112m

**2005**

Educational follow-up of children

**2015**

Time point at which primary exposures and confounders that included in the analysis were measured

T1^₮^

T2 ^¥^

^₮^assessment time-point 1, ^¥^assessment time-point 2

# Table S1: Difference beteen those children who remain and lost to follow-up from age of 6.5 to T1

| **Characteristics at age 6.5 (n=830)** | | **Had educational information at T1**^₮^ | **Had no educational information at T1** | **χ^2^ P value** |
| --- | --- | --- | --- | --- |
|  |  | **Number (%)**  **782 (94.2)** | **Number (%)**  **48 (5.8)** |  |
| Maternal CMD | Low SRQ (<6) | 749 (94.5)  33 (89.2) | 44 (5.5)  4 (10.8) | 0.180 |
|  | High SRQ (≥6) |  |  |  |
| Maternal literacy | Literate | 107 (89.9)  675 (94.9) | 12 (10.1)  36 (5.1) | 0.030 |
|  | Non-literate |  |  |  |
| Paternal literacy | Literate | 481 (94.0)  275 (95.2) | 31 (6.0)  14 (4.8) | 0.475 |
|  | Non-literate |  |  |  |
| Had hunger in last month | No | 745 (94.3)  37 (92.5) | 45 (5.7)  3 (7.5) | 0.633 |
|  | Yes |  |  |  |
| Had emergency resources | Yes | 486 (96.2)  296 (91.1) | 19 (3.8)  29 (8.9) | 0.002 |
|  | No |  |  |  |
| Roof cover | Corrugated iron | 248 (93.6)  534 (94.5) | 17 (6.4)  31 (5.5) | 0.593 |
|  | Thatched |  |  |  |
| Nutritional status | Non-stunted | 537 (95.0)  233 (92.5) | 28 (5.0)  19 (7.5) | 0.143 |
|  | Stunted |  |  |  |
| Child sex | Girl | 384 (94.6)  398 (93.9) | 22 (5.4)  26 (6.1) | 0.660 |
|  | Boy |  |  |  |
| Birth order | Middle or last | 696 (96.4)  86 (79.6) | 26 (3.6)  22 (20.4) | <0.001 |
|  | First |  |  |  |

^₮^assessment time-point 1

# Table S2: Difference beteen those children who do and do not have educational information at T2 (2014/2015 academic year)

| **Characteristics measured at T1**^₮^ **(n=788)** | | **Had educational information at T2**^¥^ | **Had no educational information at T2** | **χ^2^ P value** |
| --- | --- | --- | --- | --- |
|  |  | **Number (%)**  **529 (67.1)** | **Number (%)**  **259(32.9)** |  |
| Maternal CMD | Low SRQ (<6) | 478 (68.0)  51 (60.0) | 225 (32.0)  34 (40.0) | 0.138 |
|  | High SRQ (≥6) |  |  |  |
| Maternal literacy | Literate | 52 (68.4)  477 (67.0) | 24 (31.6)  235 (33.0) | 0.801 |
|  | Non-literate |  |  |  |
| Paternal literacy | Literate | 330 (68.6)  182 (66.2) | 151 (31.4)  93 (33.8) | 0.493 |
|  | Non-literate |  |  |  |
| Had hunger in last month | No | 511 (67.5)  17 (56.7) | 246 (32.5)  13 (43.3) | 0.215 |
|  | Yes |  |  |  |
| Had emergency resources | Yes | 371 (69.0)  158 (63.2) | 167 (31.0)  92 (36.8) | 0.109 |
|  | No |  |  |  |
| Roof cover | Corrugated iron | 236 (69*.8)  293 (65.1) | 102 (30.2)  157 (34.9) | 0.163 |
|  | Thatched |  |  |  |
| Nutritional status | Non-stunted | 417 (76.4)  111 (63.8) | 129 (23.6)  63 (36.2) | 0.001 |
|  | Stunted |  |  |  |
| Child sex | Girl | 246 (63.7)  283 (70.6) | 140 (36.3)  118 (29.4) | 0.041 |
|  | Boy |  |  |  |
| Birth order | Middle or last | 469 (66.8)  60 (69.8) | 2133 (33.2)  26 (30.2) | 0.581 |
|  | First |  |  |  |

^₮^assessment time-point 1, ^¥^assessment time-point 2
